# Ability of ecological deprivation indices to measure social inequalities in a French cohort

**DOI:** 10.1186/s12889-017-4967-3

**Published:** 2017-12-15

**Authors:** Sofia Temam, Raphaëlle Varraso, Carole Pornet, Margaux Sanchez, Aurélie Affret, Bénédicte Jacquemin, Françoise Clavel-Chapelon, Grégoire Rey, Stéphane Rican, Nicole Le Moual

**Affiliations:** 1 INSERM, U1168, VIMA: Aging and chronic diseases. Epidemiological and public health approaches, 16 Avenue Paul-Vaillant Couturier, F-94807 VILLEJUIF, Cedex France; 2 Univ Paris Sud, Le Kremlin-Bicêtre, France; 3 Univ Versailles St-Quentin-en-Yvelines, UMR-S 1168, F-78180 Montigny le Bretonneux, France; 4 Agence Régionale de Santé (ARS) Basse-Normandie, Caen, France; 5 Centre for Research in Epidemiology and Population Health (CESP), Mode de vie, gènes et santé: épidémiologie intégrée trans-générationnelle, INSERM, U1018, Villejuif, France; 6 Centre for Research in Environmental Epidemiology (CREAL), Barcelona, Spain; 7 Universitat Pompeu Fabra (UPF), Barcelona, Spain; 8 CIBER Epidemiología y Salud Pública (CIBERESP), Barcelona, Spain; 9 INSERM, CépiDc, Le Kremlin-Bicêtre, France; 10 LADYSS, Laboratoire dynamiques sociales et recompositions des espaces, Université Paris Ouest Nanterre La Défense, Nanterre, France

**Keywords:** Socioeconomic factors, Deprivation index, Social inequalities

## Abstract

**Background:**

Despite the increasing interest in place effect to explain health inequalities, there is currently no consensus on which kind of area-based socioeconomic measures researchers should use to assess neighborhood socioeconomic position (SEP). The study aimed to evaluate the reliability of different area-based deprivation indices (DIs) in capturing socioeconomic residential conditions of French elderly women cohort.

**Methods:**

We assessed area-based SEP using 3 DIs: Townsend Index, French European Deprivation Index (FEDI) and French Deprivation index (FDep), among women from E3N (*Etude épidémiologique auprès des femmes de la Mutuelle Générale de l’Education Nationale*). DIs were derived from the 2009 French census at IRIS level (smallest geographical units in France). Educational level was used to evaluate individual-SEP. To evaluate external validity of the 3 DIs, associations between two well-established socially patterned outcomes among French elderly women (smoking and overweight) and SEP, were compared. Odd ratios were computed with generalized estimating equations to control for clustering effects from participants within the same IRIS.

**Results:**

The analysis was performed among 63,888 women (aged 64, 47% ever smokers and 30% overweight). Substantial agreement was observed between the two French DIs (Kappa coefficient = 0.61) and between Townsend and FEDI (0.74) and fair agreement between Townsend and FDep (0.21). As expected among French elderly women, those with lower educational level were significantly less prone to be ever smoker (Low vs. High; OR [95% CI] = 0.43 [0.40–0.46]) and more prone to being overweight (1.89 [1.77–2.01]) than women higher educated. FDep showed expected associations at area-level for both smoking (most deprived vs. least deprived quintile; 0.77 [0.73–0.81]) and overweight (1.52 [1.44–1.62]). For FEDI opposite associations with smoking (1.13 [1.07–1.19]) and expected association with overweight (1.20 [1.13–1.28]) were observed. Townsend showed opposite associations to those expected for both smoking and overweight (1.51 [1.43–1.59]; 0.93 [0.88–0.99], respectively).

**Conclusion:**

FDep seemed reliable to capture socioeconomic residential conditions of the E3N women, more educated in average than general French population. Results varied strongly according to the DI with unexpected results for some of them, which suggested the importance to test external validity before studying social disparities in health in specific populations.

**Electronic supplementary material:**

The online version of this article (10.1186/s12889-017-4967-3) contains supplementary material, which is available to authorized users.

## Background

There have been growing evidence that both individual and neighborhood socioeconomic position (SEP) play a role in shaping health and health inequalities [1]. However, despite the increasing interest in place effect to explain health inequalities [2], there are currently no gold standard and no consensus on which kind of area-based socioeconomic measures researchers should use to assess neighborhood SEP [3].

Various area-based socioeconomic measures, such as deprivation index (DI), have been developed using census data [4]. For example, the Townsend deprivation index [5], developed in England, has been widely applied mostly in Anglo-Saxon countries [6] but also in French studies [7]. It has been shown that the Townsend index was poorly adapted to the specific French social and economic context [8] and more overall it is recognized that DIs are specific to their country of origin [9]. Despite the growing development and used of DIs, they are rarely explicitly validated [1, 10] and how DIs are built may have important impact on its explanatory power with respect to health [11, 12]. In addition, some methodological limitations have been underlined [4]. Composite area-based SEP may be sensitive to urban-rural differences according to the items included [13]. DIs that are based on census-data often include variables related to the active population or male-centered [14] and might not be suitable to specific populations, such as elderly [15, 16] or women [17].

Recently, French specific DIs have been developed, based on different statistical methods and following different objectives [18–23]. Some of them were built as a proxy of individual SEP [24] such as the French European Deprivation Index (FEDI) [19] whereas others were built to capture health inequalities at ecological level such as the French Deprivation Index (FDep) [22].

To the best of authors’ knowledge, no study had examined both agreement between DIs and their ability to detect well-established socially dependent outcomes in the French context and more specifically among elderly women as a check on external validity. At individual-level, smoking initiation is a well-established SEP related outcome among French elderly women (more smokers among those with higher educational level) [25, 26] and a similar trend has been observed at area-level [27]. In the same way, overweight status is a known SEP related outcome at individual level (less overweight women among those with higher educational level) [28–30] with a consistent trend at area-level [31]. In addition, Chaix et al. underlined that area-based SEP is associated in the same direction as individual SEP with smoking and overweight [32].

The main objective of our study was to investigate indirectly the ability of different DIs to measure socioeconomic residential conditions of a large population of French elderly women [33]. We tested the external validity of these DIs, as previously performed [34, 35], by comparing their ability to demonstrate expected associations with smoking and overweight status.

## Methods

### Study population

The E3N cohort (Epidemiological prospective cohort study among women of the Mutuelle Générale de l’Education Nationale), was initiated in 1990 to study major chronic diseases, among 98,995 women, born between 1925 and 1950, and insured under a health insurance plan covering mostly teachers [33]. Questionnaires are sent, roughly every 2 years, to update information on lifestyle factors and newly diagnosed medical conditions. E3N was approved by the French Commission for Data Protection and Privacy.

### Indicators of socioeconomic status

The individual-level SEP was evaluated using the women’s educational level in 4 classes (<high school diploma, high school to 2-level university, 3−/4-level and 5-level) collected in 1990.

We calculated 3 DIs (Additional file 1): the Townsend index [5], FEDI [19] and FDep [22] using the 2009 French national census at IRIS level (regrouped statistical information blocks). IRIS is the smallest geographical division in France with 2000 inhabitants in average (towns with more than 5000 inhabitants are divided into several IRIS, while smaller towns form one IRIS each). The homogeneity of each unit is based mainly on habitat type (residential area, public housing, etc.). The Townsend Index is a combination of 4 census-derived variables. In the present study, the proportion of primary residences with more than 1 person per room was used instead of the percentage of overcrowded households (not available in France). FEDI is a combination of 10 weighted census-derived variables associated to average social deprivation in France and identified to best represent individual experience of deprivation [19]. FDep is generated using principal component analysis (PCA) from a set of 4 census-derived variables with both negative and positive socioeconomic dimensions (Additional file 1: Table S1).

We calculated the DIs for 44,709 Metropolitan French IRIS for which the census-derived variables were available (i.e. 89.1%; due to data confidentiality, median income was not available in 5481 IRIS with less than 50 households). We ranked the DIs score (using the population-weighted approach) into five deprivation quintiles from the least (Q1) to the most (Q5) deprived IRIS with approximately 20% of the French population in each ones. The population-weighted approach classify the proportion of the deprived population rather than of the deprived areas [36]. We applied the score and the quintiles of each DI to the women’s residential address in 2005 previously geocoded by a commercial firm, which attributed for each address a level of geocoding accuracy. Geocoding was considered as “precise” if the exact address was found automatically with the highest possible precision (<15 m).

### Outcomes & covariables

We used the smoking status in two classes (ever smokers vs. never smokers) to evaluate retrospectively smoking initiation [25]. We defined the overweight status using the body mass index (weight(kg)/height(m)^2^) with a cut-off at 25 kg/m^2^. Both outcomes were reported by participants in the questionnaire sent in 2005.

To take into account the impact of urban-rural settings on the DIs, we classified the addresses using the degree of urbanicity, based on the concept of urban unit, defined at commune-level by INSEE (French National Institute of Statistics and Economic Studies) [22]. An urban unit is a town or a group of towns that includes at least 2000 inhabitants and in which no building is farther than 200 m away from its nearest neighborhood. The degree of urbanicity is defined in 4 classes according to the population size: “Paris-and-suburbs”, “urban” (100,000 to 1,999,999 inhabitants), “quasi-urban” (10,000–99,999), “quasi-rural and rural” (<9999).

### Strategy of analysis

Descriptive analyses were performed by t-tests for continuous variables and chi-squared tests for categorical variables. We quantified the degree of agreement between the 3 DIs in classifying the women into the same or a close quintile using weighted Cohen’s Kappa (Kw) statistics (0.81–1.00:almost perfect agreement, 0.61-0.80:substantial, 0.41–0.60:moderate, 0.21–0.40:fair, 0.00–0.20:slight, <0:poor agreement) [37]. We compared the mean of the DIs (as continuous variables) of each educational level. We hypothesized that the better DI, for our specific French elderly women population, would be the one that was associated with well-known socially patterned outcomes.

We evaluated the ability to detect well-established associations (external or convergent validity) as previously performed for SEP [34, 35] and occupational exposures [38]. We studied the associations between each SEP indicators and both smoking and overweight status using logistic regressions models adjusted for age (Software package SAS 9.3). The reference category for the SEP indicators was the highest educational level and the least deprived quintile (Q1). The odd ratio estimates were computed with generalized estimating equations (SAS GENMOD procedure) to control for clustering effects from participants within the same IRIS in a context of sparse clustered data (87% of IRIS contained less than 5 participants, Table 1) [39].

**Table 1:** Proportion of participants by IRIS size and proportion of IRIS by size

	All	Urban	Rural
Total number of participants	63,888	49,107	14,781
Number of participants by IRIS			
Mean ± SD	2.9 ± 2.5	3.5 ± 2.8	1.8 ± 1.5
Median	2	3.0	1.0
Min - Max	1–45	1–45	1–18
Number of participants by IRIS size, n (%)^1^			
IRIS with 1 participant	8975 (14.0)	3680 (7.5)	5295 (35.8)
IRIS with 2 participants	9056 (14.2)	5826 (11.9)	3230 (21.9)
IRIS with 3 to 5 participants	22,750 (35.6)	18,608 (37.9)	4142 (28.0)
IRIS with 6 to 10 participants	17,403 (27.2)	15,638 (31.8)	1765 (11.9)
IRIS with 11 to 20 participants	5372 (8.4)	5023 (10.2)	349 (2.4)
IRIS with 21 to 45 participants	332 (0.5)	332 (0.7)	0 (−)
Number of IRIS, n (%) ^2^	22,372	14,036	8336
IRIS with 1 participant	8975 (40.1)	3680 (26.2)	5295 (63.5)
IRIS with 2 participants	4528 (20.2)	2913 (20.8)	1615 (19.4)
IRIS with 3 to 5 participants	6052 (27.1)	4895 (34.9)	1157 (13.9)
IRIS with 6 to 10 participants	2394 (10.7)	2153 (15.3)	241 (2.9)
IRIS with 11 to 20 participants	409 (1.8)	381 (2.7)	28 (0.3)
IRIS with 21 to 45 participants	14 (0.1)	14 (0.1)	0 (−)

*±SD* Standard deviation

*IRIS* Regrouped statistical information blocks

The denominator corresponds to ^1^ total number of participants, ^2^ number of IRIS

More prevalent smoking initiation [25, 26] and less prevalent overweight [28–30] are well-established associations described in the literature among French elderly women with higher educational level. In addition at area-level, risk factors associated to lifestyle (such as smoking and overweight) are expected to be in the same direction as individual SEP due to normative standards and behavioral characteristics [32]. Therefore, the present analysis relied on the following assumptions: (i) more prevalent smoking and less prevalent overweight among elderly women living in less deprived area; (ii) no association between Townsend index and both outcomes, as this index was not adapted to the French context (iii) The French DIs were not built in the same way (neither for the same purpose) and might classify differently the E3N women regarding their residential deprivation context. We aimed to evaluate, which one, if any, may be more adapted to capture socioeconomic residential conditions of our specific elderly women population (in average, highly educated compared to the general population of the same age group).

We performed several sensitivity analyses. First, to test the robustness of the DIs [3], we stratified the analyses on (i) age (<65 vs. ≥65 years) to check the hypothesis of stronger associations among older women for smoking; (ii) geocoding accuracy to check the hypothesis of stronger associations among those with precise geocoding. Furthermore, to assess the impact of urban-rural settings on the DIs as previously suggested [8], we adjusted on degree of urbanicity. Furthermore, individual SEP was evaluated at baseline and DIs in 2009 (census data not available at baseline) which may induce misclassification bias. Therefore, we performed a sensitivity analysis including only women who did not move between 1991 and 2005 (*n* = 39,130; 61%). Finally, we performed analyses using the un-weighted approach (i.e. quintile ranked independently of the population size).

## Results

### Description of the study population

Out of 71,411 women who completed the questionnaire in 2005, we excluded 10.6% from the analyses due to missing data on IRIS (*n* = 462), DIs (*n* = 424), educational level (*n* = 2824), smoking status (*n* = 1115) and body mass index (*n* = 2698) (Additional file 1: Fig. S1). The excluded women were slightly older, more smokers during life, more educated and overweight (not shown). The final study population included 63,888 women. The women (Table 2) were in average 64 years old, 47% had ever smoked and 30% were overweight. A majority had attended at least the high school diploma (88%) and lived in urban or quasi-urban areas (77%). The older women (≥ 65 years old) had significantly lower educational level, were less often ever smokers and more often overweight compared to the younger. In average, FDep did not vary according to age (*p* = 0.30) contrary to FEDI and Townsend (*p* < 0.0001).

**Table 2:** Description of the study population, overall and stratified by age

	All	<65 years old	≥65 years old	Overall *p*-value (crude)†
*N*	63,888	36,975	26,913	
Age, mean ± SD	64.4 ± 6.4	59.8 ± 2.7	70.8 ± 4.1	
Ever smokers	46.9	51.0	41.1	
Overweight status	30.0	28.2	32.6	<0.0001
Individual-educational level				
< High school	11.7	9.3	14.9	<0.0001
High school to 2-level university	51.2	48.4	55.1	
3−/4-level university	18.8	23.3	12.6	
5-level university	18.3	18.9	17.5	
Degree of urbanicity				
Paris and suburbs	10.2	9.6	11.0	<0.0001
Urban	33.0	30.6	36.3	
Quasi-urban	33.7	34.8	32.1	
Quasi-rural and Rural	23.1	25.1	20.5	
Area-level SEP, mean ± SD				
FDep	−0.3 ± 1.0	−0.3 ± 1.0	−0.3 ± 1.0	0.30
Min-Max	−4.1 – 3.3	−4.1 – 3.3	−4.1 – 3.2	
FEDI	−0.3 ± 3.4	−0.4 ± 3.5	−0.1 ± 3.4	<0.0001
Min-Max	−8.7 – 28.5	−8.7 – 28.5	−8.2 – 25.6	
Townsend	1.2 ± 3.1	1.0 ± 3.1	1.4 ± 3.1	<0.0001
Min-Max	−8.9 – 14.3	−8.9 – 14.3	−7.1 – 14.0	

Data are presented as %, unless otherwise stated

†: t-tests were used for continuous variables and Chi-squared tests were used for categorical variables

Overweight corresponds to a Body Mass Index ≥25 kg/m^2^

*FDep* French Deprivation index, *FEDI* French European Deprivation Index

The Deprivation indices are presented in continuous

Degree of urbanicity is a geographic measure of population density, defined at commune-level by INSEE (French National Institute of Statistics and Economic Studies): rural and quasi-rural (<10,000 inhabitants), quasi-urban (from 10,000 to 99,999), urban (from 100,000 to 1,999,999) and Paris-and-suburbs (Paris Urban Unit)

### Individual characteristics, individual SEP and DIs

Women who lived in urban areas were significantly older, ever smokers and less overweight compared to women living in rural areas (Table 3). The FDep index tended to decrease with the increasing of urbanicity (especially for Paris and suburbs). On the contrary, FEDI and Townsend increased with the increasing of urbanicity, i.e. in urban areas, women were classified as living in more deprived neighborhoods. As expected, when the whole France DIs quintiles were applied to the E3N population, less than 15% of the women were living in the most deprived areas (Q5; Additional file 1: Fig. S2). We observed significant linear associations between educational level and the scores of the DIs (Table 4). FDep and FEDI decreased when educational level increased with a clearer linear trend for FDep whereas an opposite trend was observed for Townsend.

**Table 3:** Individual characteristics and deprivation indices according to the degree of urbanicity

	All	Rural and quasi-rural	Quasi- urban	Urban	Paris and suburbs	Overall *p*-value (crude)
*N*	63,888	14,781	21,531	21,080	6496	
Age ≥ 65 years old	42.1	37.4	40.2	46.4	45.6	<0.0001
Ever smokers	46.9	45.3	45.5	46.8	55.5	<0.0001
Overweight	30.0	32.9	29.6	29.0	28.1	<0.0001
Individual SEP						
Higher educational level^a^	18.3	9.8	17.6	19.0	38.1	<0.0001
Area-level SEP, mean ± SD						
FDep	−0.34 ± 1.0	0.08 ± 0.6	−0.6 ± 0.9	−0.1 ± 0.9	−1.5 ± 1.2	<0.0001
FEDI	−0.26 ± 3.4	−1.2 ± 2.4	−1.3 ± 3.3	1.0 ± 3.4	1.1 ± 4.2	<0.0001
Townsend	1.18 ± 3.1	−0.88 ± 1.7	0.2 ± 2.4	2.4 ± 2.6	5.3 ± 3.2	<0.0001

Data are presented as %, unless otherwise stated

Degree of urbanicity is a geographic measure of population density, defined at commune-level by INSEE (French National Institute of Statistics and Economic Studies): rural and quasi-rural (<10,000 inhabitants), quasi-urban (from 10,000 to 99,999), urban (from 100,000 to 1,999,999) and Paris and suburbs (Paris Urban Unit)

*SEP* Socioeconomic position, *FDep* French Deprivation index, *FEDI* French European Deprivation Index. The Deprivation index variables are presented in continuous

Overweight corresponds to a Body Mass Index ≥25 kg/m^2^

^a^5-level university French diploma

**Table 4:** Mean of DIs scores by individual educational level

	n	FDep	FEDI	Townsend
Educational level				
< High school	7454	−0.16 (−0.17; −0.14)	0.11 (0.06; 0.17)	1.26 (0.21; 1.31)
High school to 2-level university	32,723	−0.23 (−0.24; −0.22)	−0.12 (−0.1; −0.08)	1.11 (1.08; 1.14)
3−/4-level university	12,008	−0.44 (−0.45; −0.42)	−0.22 (−0.27; −0.18)	1.44 (1.39; 1.49)
5-level university	11,703	−0.73 (−0.75; −0.71)	−0.22 (−0.26; −0.17)	2.00 (1.95; 2.05)
p-value for trend		<0.0001	<0.0001	<0.0001

Least squares means (95% confidence interval) from generalized estimating equation (GEE) methods

*FDep* French Deprivation index, *FEDI* French European Deprivation Index

N.B.: a lower DI score means less deprivation

The agreement was substantial between the two French DIs (κw = 0.61), whatever the degree of urbanicity, except for Paris and suburbs (0.28). The agreement between Townsend and FEDI was substantial (0.74) and fair with FDep (0.21).

### External validity

**Fig. 1:**
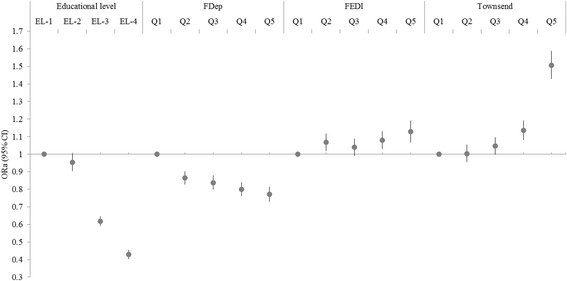
Associations between individual educational level and three area-based deprivation indices with smoking status. ORa (95% CI) = odd ratio adjusted for age (95% confidence interval) from generalized estimating equation (GEE) methods. Educational level (EL) was categorized in 4 classes (EL-1: 5-level university; EL-2: 3−/4-level university diploma; EL-3: high school to 2-level university diploma; EL-4: ≤ high school diploma), with 5-level university diploma as the reference. FDep: French Deprivation index, FEDI: French European Deprivation Index. Q1: least deprived quintile (reference); Q5: most deprived quintile. Smoking status was defined as ever-smoker (ref) vs. never smoker. *p*-values for trend were significant (<0.0001) for the four indicators

**Fig. 2:**
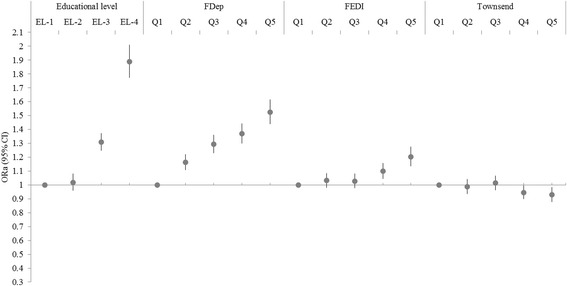
Associations between individual educational level and three area-based deprivation indices with overweight status. ORa (95% CI) = odd ratio adjusted for age (95% confidence interval) from generalized estimating equation (GEE) methods. Educational level (EL) was categorized in 4 classes (EL-1: 5-level university; EL-2: 3−/4-level university diploma; EL-3: high school to 2-level university diploma; EL-4: ≤ high school diploma), with 5-level university diploma as the reference. FDep: French Deprivation index, FEDI: French European Deprivation Index. Q1: least deprived quintile (reference); Q5: most deprived quintile. Overweight status was defined as a Body Mass Index <25 kg/m^2^ (ref) vs. ≥25 kg/m^2^. *p*-values for trend were significant (<0.01) for the four indicators

As expected in this French elderly women population, educational level was associated with smoking with a significant trend (Fig. 1). Women with lower educational level were less prone to be ever smokers, compared to those higher educated (Low vs. High; OR [95% CI] = 0.43 [0.40; 0.46], (p for trend <0.0001). At area-level, only FDep showed the same pattern (i.e., women living in more deprived areas were less prone to be ever smokers (most deprived (Q5) vs. least deprived quintile (Q1); 0.77 [0.73; 0.82], p for trend <0.0001). We observed opposite significant associations using FEDI (1.13 [1.07; 1.20]) and Townsend (1.51 [1.43; 1.59]). Regarding overweight status (Fig. 2), as expected, women with lower educational level were significantly more often overweight (1.89 [1.77; 2.01]), p for trend <0.0001) compared to women higher educated. We observed a similar pattern with both FDep (1.52 [1.44; 1.62] and FEDI (1.20 [1.13; 1.28]), and an opposite association with Townsend (0.93 [0.88; 0.99]).

### Sensitivity analyses

We observed stronger associations among older women (≥ 65 years old) for smoking (Additional file 1: Fig. S3). When we restricted the analysis to women with the better geocoding accuracy (50% of the population), we observed stronger associations for both smoking and overweight status (not shown). Analyses adjusted on degree of urbanicity showed similar results with FDep for both smoking and overweight whereas associations became non-significant for smoking and stronger for overweight when using FEDI (Table 5). Similar results were also observed when the analysis was restricted to non-movers between 1991 and 2005 (*n* = 39,130; 61%) (Additional file 1: Table S2). Finally, weighted quintiles ranking method gave slightly stronger associations with both smoking and overweight status, compared to unweighted ones, especially for FDep (Additional file 1: Fig. S4-S5).

**Table 5:** Associations between area-based deprivation indices with smoking and overweight adjusted on degree of urbanicity

	Smoking			Overweight
	All^a^	Age < 65	Age ≥ 65	All^a^
n	63,888	36,975	26,913	63,888
FDep				
Quintile 1 [ref.]	–	–	–	–
2	0.90 (0.86; 0.94)	0.96 (0.90; 1.02)	0.83 (0.77; 0.89)	1.13 (1.08; 1.19)
3	0.89 (0.85; 0.93)	0.94 (0.88; 1.00)	0.83 (0.78; 0.89)	1.24 (1.18; 1.31)
4	0.85 (0.81; 0.90)	0.94 (0.88; 1.01)	0.74 (0.68; 0.80)	1.31 (1.24; 1.38)
5	0.80 (0.75; 0.84)	0.84 (0.78; 0.91)	0.74 (0.68; 0.81)	1.49 (1.41; 1.58)
p-value for trend	<0.0001	<0.0001	<0.0001	<0.0001
FEDI				
Quintile 1 [ref.]	–	–	–	–
2	1.05 (1.00; 1.10)	1.05 (0.99; 1.12)	1.05 (0.97; 1.13)	1.05 (1.00; 1.10)
3	1.01 (0.96; 1.06)	1.06 (1.00; 1.13)	0.94 (0.87; 1.01)	1.06 (1.01; 1.12)
4	1.02 (0.98; 1.08)	1.06 (1.00; 1.13)	0.97 (0.90; 1.05)	1.17 (1.11; 1.23)
5	1.04 (0.98; 1.10)	1.07 (0.99; 1.16)	0.99 (0.90; 1.08)	1.33 (1.25; 1.41)
p-value for trend	0.45	0.04	0.21	<0.0001
Townsend				
Quintile 1 [ref.]	–	–	–	–
2	0.99 (0.94; 1.04)	1.00 (0.94; 1.06)	0.98 (0.90; 1.06)	1.02 (0.97; 1.08)
3	1.01 (0.96; 1.06)	1.02 (0.96; 1.08)	1.00 (0.92; 1.08)	1.09 (1.04; 1.15)
4	1.07 (1.01; 1.13)	1.13 (1.05; 1.21)	1.00 (0.92; 1.08)	1.07 (1.01; 1.14)
5	1.39 (1.31; 1.48)	1.41 (1.30; 1.53)	1.35 (1.23; 1.49)	1.11 (1.03; 1.19)
p-value for trend	<0.0001	<0.0001	<0.0001	0.001

Data are presented as OR (95% confidence interval) adjusted on degree of urbanicity (^a^and adjusted on age) from generalized estimating equation (GEE) methods

Degree of “urbanicity”, a geographic measure of population density, defined at commune-level by INSEE (French National Institute of Statistics and Economic Studies): rural and quasi-rural (<10,000 inhabitants), quasi-urban (from 10,000 to 99,999), urban (from 100,000 to 1,999,999) and Paris-and-suburbs (Paris Urban Unit)

*FDep* French Deprivation index, *FEDI* French European Deprivation Index

Q1: least deprived (reference); Q5: most deprived

## Discussion

Our findings showed substantial agreement between the two French area-based DIs and between Townsend and FEDI but fair agreement between Townsend and FDep. We observed expected known associations among French elderly women between individual educational level and both smoking and overweight. At area-level, only FDep showed similar patterns for both smoking and overweight. Inconsistent associations were observed for the two others DIs. FDep seemed reliable to capture socioeconomic residential conditions of the E3N elderly women, highly educated and living mostly in urban areas.

### Ability of FDep to predict outcomes with well-known social patterns

The ability of area-based indicators to predict known socially patterned outcomes have been previously studied mostly in Anglo-Saxon countries [40]. Our study, the first one performed in a very large French epidemiological cohort, showed the reliability of FDep to capture socioeconomic residential conditions among E3N elderly women. Results for FDep were consistent with those of the literature for both smoking and overweight status with a clear gradient between the quintiles [27, 31]. Moreover, we observed a stronger association among older women between FDep and smoking, similarly to what was observed with educational level. It has been suggested that commonly used DIs suited poorly to study inequalities in older people especially because they included variables related to the active population or male-centered (social class) [14]. The use of a DI in our population could therefore be a limitation. However, interestingly, FDep did not varied according to age, contrary to FEDI and Townsend for which older women were classified as more deprived in average. Stronger associations were also observed with FDep when we restricted the analyses to women with precise geocoding linkage, which was expected in the case of non-differential geocoding errors regarding SEP. [41] In French ecological studies, stronger associations were observed with FDep compared to Townsend, studying DIs and all-cause mortality at commune-level [22], consistently to our results. In addition, FDep was found less sensitive to urban-rural differences than Townsend, studying associations with colorectal cancer screening attendance [8] consistently to our results.

### Interpretation of the differences between FDep, Townsend and FEDI

As expected, Townsend was not adapted to evaluate residential deprivation in a French context [20, 22]. Although FDep and FEDI showed a substantial agreement in classifying the women across the range of deprivation, we observed discrepancies in predicting smoking and overweight social patterns in E3N. This discrepancy could be explained by their different mode of construction. FDep was constructed to maximize the heterogeneity of the components using a PCA [22]. FEDI was composed of weighted variables identified to best represent individual experience of deprivation and based on average social deprivation [19] and thus might be less adapted to capture the variety of socio-spatial situations that composed the French territory. Moreover, DIs we applied here have not been created in the same context. FEDI was constructed to proxy individual SEP whereas FDep was setup in the context of ecological approaches.

The different items included in the DIs may also explain the disagreement. For example, FEDI and Townsend included the "proportion of households not owner occupied", "primary residence with more than 1 person per room" and “without a car”. These items are known to vary according to the degree of urbanicity and specifically in rural vs. urban areas [13, 42]. For example, in rural areas, not possessing cars could be an obstacle for mobility and though be a proxy of deprivation, whereas in urban areas, especially in large cities, it is common to have no car as public transport is particularly developed in France. Likewise, overcrowding and home-ownership are not comparable between urban and rural settings and could be a marker of deprivation in rural areas but not always in urban ones. We observed that the FDep index tended to decrease (i.e. less deprivation) with the increasing of urbanicity, especially for Paris and suburbs. On the contrary, FEDI and Townsend increase (i.e. more deprivation) with the increasing of urbanicity. This opposite trend which appeared clearly on the maps of the distribution of the IRIS (Additional file 1: Fig. S6), could ensue from these items. For example, the map with the Townsend index showed light shades because the majority of the French territory is rural. On the contrary, the map with the FDep index is darker because rural areas are classified as more deprived than urban ones. Furthermore, individual characteristics of the participants varied according to the degree of urbanicity of their place of residence, with higher prevalence of ever smokers and less prevalence of overweight in urban areas that might also explain the unexpected associations observed for Townsend and FEDI with smoking.

### Strengths and limitations

Our study presented several strengths. We used a very large population sample homogeneously distributed across the French territory. At area-level, composite indicators were more effective to take into account the multidimensionality of the SEP than a single one [4]. Our results confirmed that FDep, initially developed at commune-level [22], was also able to capture the inter- and intra-urban socio-spatial divisions existing in France at IRIS level. In addition, we used the population-weighted approach to construct the DIs quintiles, which allowed a better classification of population and gave stronger associations between FDep and the outcomes. To the best of authors’ knowledge the present epidemiological study is the first one to compare weighted and un-weighted methods. We tested two different outcomes with established social pattern, smoking and overweight, to assess the robustness of the DIs [3]. We used GEE models to control for clustering effects from participants within the same IRIS in a context of sparsely clustering data, as recommended [39]. We used the finest spatial unit with socio-demographic data available in France to minimize misclassification and potential ecological bias as recommended [43]. Associations observed between FDep and known SEP related outcomes fit within 2 a priori criteria described as external validity and robustness by Krieger et al. [3].

The study nevertheless had some limitations. Models including both individual and area-level variables were not performed in the present study because the hypotheses were based on studies using either SEP indicators at individual- or at area-level separately. Therefore, we were not able to distinguish compositional from contextual effects. Women’s residential history were not taken into account. However, less than 30% of movers were identified between 1991 and 2005 in a sub-E3N population [44]. E3N women were 45 years at baseline, thus we hypothesized that their social trajectory was already settled and did not change much during this period. In addition, census data were not available to calculate the DIs at baseline. However, French studies have shown that spatial distribution of deprivation did not change substantially since 1991 [45, 46]. Nonetheless, we performed a sensitivity analysis including only women who did not move and the conclusion was similar.

### Choosing the most appropriate contextual indicators to capture socioeconomic conditions in a specific population

Historically, area-based SEP has been used as a surrogate of individual-SEP in medical records [47], but this strategy have been questioned particularly in Anglo-Saxon countries [48]. Some methodological studies have compared the agreement between individual and area-based SEP and their ability in predicting health outcomes [35, 49] with conflicting results. Poor agreement has been reported between self-reported individual income and area-based income [49]. While, in others studies, area-based SEP was considered as a good proxy of individual-level SEP [35] allowing the prediction of socially patterned outcomes. Finally, it has been underlined that area-based SEP indicators fairly classify socially homogenous areas (most and least deprived neighborhoods) but failed sometimes to classify the in-between situations that are more heterogeneous [50]. In the relatively highly educated E3N population, we observed a clear gradient across the quintiles of FDep for both outcomes, whatever the strategy of analysis. The E3N population is not representative of the French elderly women. They have in average higher educational level than French elderly women and probably healthier conditions. However, even in this specific population, we found that social disparities in smoking and overweight do not affect only extreme social situations but rather the socioeconomic gradient [43]. Our objective was to determine which area-based SEP could meaningfully be used to further study social disparities in health in an elderly women population. It has been underlined that DIs might not be suitable in specific populations, such as elderly [15] or women [15, 17]. FDep appeared to be a good indicator to capture inter- and intra-urban socio-spatial divisions existing in France and seemed reliable to capture socioeconomic residential conditions of the E3N elderly women population, mostly teachers living in urban areas.

## Conclusion

In conclusion, we showed that associations might vary strongly according to DIs with unexpected results for some of them. Our results suggested that it is important to test external validity to found well known associations before studying social disparities in health in specific populations.

## Additional file

Additional file 1: Table S1. Variables included in each area-based deprivation index (n=44,709 IRIS). **Table S2.** Associations between individual- and area-level SEP with smoking initiation and overweight among women who did not move between 1991 and 2005 (n=39,130). **Figure S1.** Flow-chart: selection of the study population (n=63,888). **Figure S2.** Distribution of the E3N population in the whole France deprivation indices quintiles. **Figure S3.** Associations between individual-level and three area-based deprivation indices with smoking status stratified by age. **Figure S4.** Comparison of the associations between three area-based deprivations indices with smoking status according to weighted and unweighted quintiles. **Figure S5.** Comparison of associations between three area-based deprivation indices with overweight status according to weighted and unweighted quintiles. **Figure S6.** Geographical distribution of the three deprivation indices at IRIS level in France. (DOCX 922 kb)

## Abbreviations

CI: Confidence interval
DI: Deprivation index
E3N: Etude épidémiologique auprès des femmes de la Mutuelle Générale de l’Education Nationale
FDep: French Deprivation index
FEDI: French European Deprivation Index
IRIS: Îlot regroupé pour l’information statistique
OR: Odd ratio
PCA: Principal component analysis
Q: Quintile
SD: Standard deviation
SEP: Socioeconomic position
