# Supplementary material for: Ability of ecological deprivation indices to measure social inequalities in a French cohort
Source: BMC Public Health. 2017 Dec 15;17:956. doi: 10.1186/s12889-017-4967-3 (PMC5732455; doi:10.1186/s12889-017-4967-3)
Supplement: Additional file 1: Table S1. — Variables included in each area-based deprivation index (n=44,709 IRIS). Table S2. Associations between individual- and area-level SEP with smoking initiation and overweight among women who did not move between 1991 and 2005 (n=39,130). Figure S1. Flow-chart: selection of the study population (n=63,888). Figure S2. Distribution of the E3N population in the whole France deprivation indices quintiles. Figure S3. Associations between individual-level and three area-based deprivation indices with smoking status stratified by age. Figure S4. Comparison of the associations between three area-based deprivations indices with smoking status according to weighted and unweighted quintiles. Figure S5. Comparison of associations between three area-based deprivation indices with overweight status according to weighted and unweighted quintiles. Figure S6. Geographical distribution of the three deprivation indices at IRIS level in France. (DOCX 922 kb) [file 12889_2017_4967_MOESM1_ESM.docx]

**Supplementary Material**

**Ability of ecological deprivation indices to measure social inequalities in a French cohort**

**Authors:**

Sofia Temam^1,2,3^, Raphaëlle Varraso^1,3^, Carole Pornet^4^, Margaux Sanchez^1,2,3^, Aurélie Affret^2,5^, Bénédicte Jacquemin^1,3,6,7,8^ Françoise Clavel-Chapelon^2,5^, Grégoire Rey^9^, Stéphane Rican^10^, Nicole Le Moual^1,3^

^1^ INSERM, U1168, VIMA: Aging and chronic diseases. Epidemiological and public health approaches, Villejuif, France

^2^ Univ Paris Sud, Le Kremlin-Bicêtre, France

^3^ Univ Versailles St-Quentin-en-Yvelines, UMR-S 1168, F-78180, Montigny le Bretonneux, France

^4^ Agence Régionale de Santé (ARS) Basse-Normandie, Caen, France

^5^ INSERM, U1018, Centre for Research in Epidemiology and Population Health (CESP), *Mode de vie, gènes et santé: épidémiologie intégrée trans-générationnelle*, Villejuif, France

^6^ Centre for Research in Environmental Epidemiology (CREAL), Barcelona, Spain

^7^ Universitat Pompeu Fabra (UPF), Barcelona, Spain

^8^ CIBER Epidemiología y Salud Pública (CIBERESP), Barcelona, Spain

^9^ INSERM, CépiDc, Le Kremlin-Bicêtre, France

^10^ Université Paris Ouest Nanterre La Défense, LADYSS, Laboratoire dynamiques sociales et recompositions des espaces, Nanterre, France

*** Corresponding author:**

Sofia Temam: [sofia.temam@inserm.fr](mailto:sofia.temam@inserm.fr)

Table S1 Variables included in each area-based deprivation index (n=44,709 IRIS)

|  | FDep | FEDI | Townsend |
| --- | --- | --- | --- |
|  |  |  |  |
| Distribution |  |  |  |
| Mean ±SD | -0.1 ±0.8 | 0.0 ±4.1 | 0.02 ±3.2 |
| Min-Max | -4.2 – 4.6 | -11.7 – 51.1 | -8.9 – 19.3 |
|  |  |  |  |
| Census-derived variables included |  | (weight) |  |
| Unemployment | x | x (0.47) | x |
| Blue-collar workers and employees in the labour force | x | x (0.37) | - |
| Median income per consumption unit or household | x | - | - |
| Second-year university level (≥ 15 years and older) | x | - | - |
| Single-parent household | - | x (0.41) | - |
| Foreigners in total population | - | x (0.23) | - |
| Low level of education (≥ 15 years and older) | - | x (0.19) | - |
| Households without a car | - | x (0.52) | x |
| Households not owner occupied | - | x (0.55) | x |
| Households without access to central or electric heating | - | x (0.34) | - |
| Primary residences with >1 person/room | - | x (0.11) | x |
| Households with ≥ 6 occupants persons |  | x (0.45) | - |

±SD= standard deviation

FDep=French Deprivation index, FEDI=French European Deprivation Index (variables are weighted)

Table S2: Associations between individual- and area-level SEP with smoking initiation and overweight among women who did not move between 1991 and 2005 (n=39,130)

|  | Smoking  OR (95%CI) | Overweight  OR (95%CI) |
| --- | --- | --- |
| Educational level |  |  |
| 5-level university [ref.] | - | - |
| 3-/4-level university | 0.96 (0.90-1.03) | 1.01 (0.94-1.09) |
| High school to 2-level university | 0.61 (0.58-0.64) | 1.31 (1.23-1.39) |
| < High school | 0.42 (0.39-0.45) | 1.80 (1.66-1.95) |
| p-value for trend | <0.0001 | <0.0001 |
| FDep |  |  |
| Quintile 1 [ref.] | - | - |
| Q2 | 0.85 (0.80-0.90) | 1.14 (1.07-1.21) |
| Q3 | 0.83 (0.78-0.88) | 1.26 (1.18-1.34) |
| Q4 | 0.79 (0.75-0.84) | 1.29 (1.20-1.37) |
| Quintile 5 | 0.73 (0.68-0.78) | 1.44 (1.34-1.55) |
| p-value for trend | <0.0001 | <0.0001 |
| FEDI |  |  |
| Quintile 1 [ref.] | - | - |
| Q2 | 1.01 (0.95-1.07) | 1.01 (0.95-1.08) |
| Q3 | 1.03 (0.97-1.10) | 1.04 (0.98-1.11) |
| Q4 | 1.05 (0.99-1.11) | 1.09 (1.02-1.16) |
| Quintile 5 | 1.07 (0.99-1.14) | 1.17 (1.08-1.26) |
| p-value for trend | 0.03 | <0.0001 |
| Townsend |  |  |
| Quintile 1 [ref.] | - | - |
| Q2 | 1.01 (0.95-1.08) | 0.98 (0.92-1.05) |
| Q3 | 1.07 (1.01-1.14) | 1.00 (0.94-1.07) |
| Q4 | 1.14 (1.08-1.22) | 0.95 (0.89-1.02) |
| Quintile 5 | 1.52 (1.41-1.63) | 0.95 (0.88-1.02) |
| p-value for trend | <0.0001 | 0.11 |

OR (95% CI) = odd ratio (95% confidence interval), adjusted for age, with SAS GENMOD procedure

Smoking was defined as ever-smoker (ref) vs. never smoker

Overweight status was defined as a Body Mass Index <25kg/m² (ref) vs. ≥25kg/m²

FDep=French Deprivation index, FEDI=French European Deprivation Index

Q1=least deprived quintile (reference); Q5=most deprived quintile.

Figure S1 Flow-chart: selection of the study population (n=63,888)

Figure S2 Distribution of the E3N population in the whole France deprivation indices quintiles

Study population

n=63,888

**Missing values**

- Residential addresses (n=462)

i.e. 302 women were living outside France and 160 addresses could not be geocoded

- Area-based deprivation indices (n=424)

- Educational level (n=2824)

- Smoking status (n=1115)

- Body Mass Index (n=2698)

Women who completed the 2005 questionnaire

n=71,411

Quintiles weighted by IRIS population

Unweighted quintiles

IRIS = *regrouped statistical information blocks*
 Weighted quintiles contain approximately 25% of the French population in each ones;
 unweighted quintiles contain approximately 25% of the score distribution

FDep=French Deprivation index, FEDI=French European Deprivation Index

Q1=least deprived; Q5=most deprived

Figure S3 Associations between individual-level and three area-based deprivation indices with smoking status stratified by age

p =0.001

p <.0001

p <.0001

p <.0001

p <.0001

p =0.42

p <.0001

p <.0001

OR (95% CI) = odd ratios (95% confidence intervals)

Smoking status: ever-smokers (ref) *vs.* never smokers

FDep=French Deprivation index, FEDI=French European Deprivation Index

Educational level (EL) was categorised in 4 classes (EL-1=5-level university; EL-2=3-/4-level university diploma; EL-3= high school to 2-level university diploma; EL-4=<high school diploma), with 5-level university diploma as the reference

Q1=least deprived (reference); Q5=most deprived. P-values on the figure are p-values for trend

Figure S4 Comparison of the associations between three area-based deprivations indices with smoking status according to weighted and unweighted quintiles

p <.0001

p <.0001

p <.0001

p <.0001

p <.0001

p <.0001

ORa (95% CI) = odd ratios adjusted for age (95% confidence intervals)

Smoking status: ever-smokers (ref) *vs.* never smokers

Weighted quintiles: contains approximately 25% of the French population in each ones; unweighted quintiles contain approximately 25% of the score distribution

FDep=French Deprivation index, FEDI=French European Deprivation Index

Q1=least deprived (reference); Q5=most deprived

P-values on the figure are p-values for trend

Figure S5 Comparison of associations between three area-based deprivation indices with overweight status according to weighted and unweighted quintiles

p <.0001

p <.0001

p <.0001

p=0.03

..0

p=0.004

p <.0001

ORa (95% CI) = odd ratios adjusted for age (95% confidence intervals)

Overweight status was defined as a Body Mass Index <25kg/m² (ref) *vs.* >=25kg/m²

Weighted quintiles contain approximately 25% of the French population in each ones; unweighted quintiles contain approximately 25% of the score distribution

FDep=French Deprivation index, FEDI=French European Deprivation Index

Q1=least deprived (reference); Q5=most deprived

P-values on the figure are p-values for trend

Figure S6 Geographical distribution of the three deprivation indices at IRIS level in France

FEDI

Townsend Index

FDep


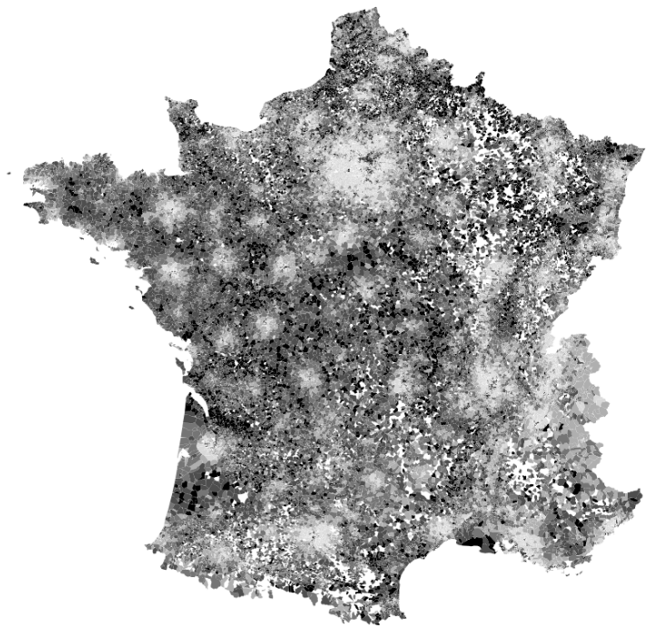

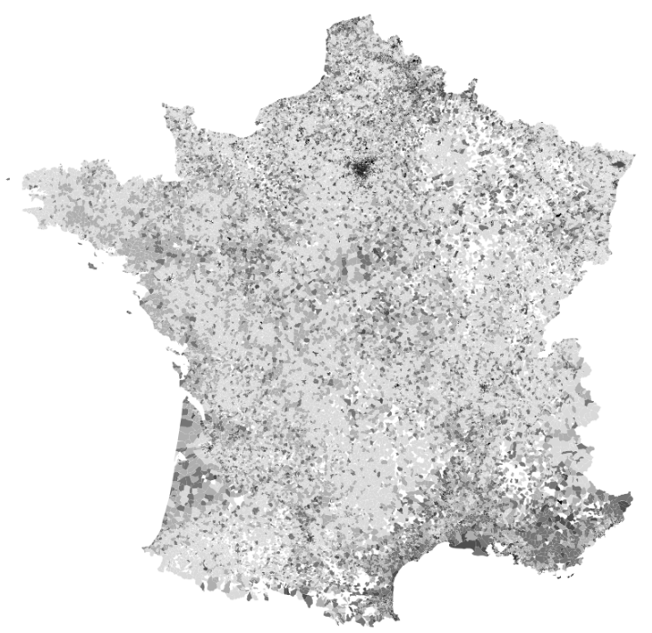

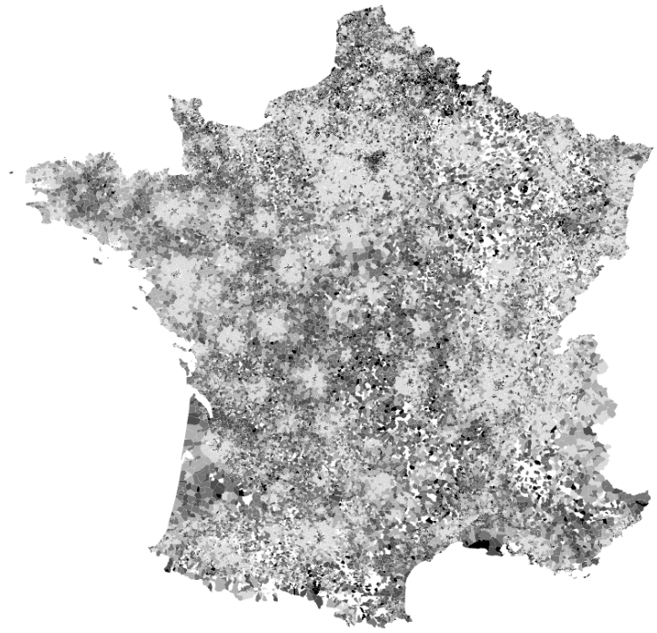


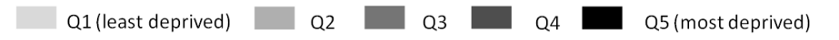


IRIS = regrouped statistical information blocks

FDep=French Deprivation index, FEDI=French European Deprivation Index

Quintiles (Q) are weighted by the IRIS population

Communes with at least 5000 inhabitants are divided in IRIS containing on average 2000 inhabitants, while smaller towns form one IRIS each.
